# Harm Reduction in the Field: First Responders’ Perceptions of Opioid Overdose Interventions

**DOI:** 10.5811/westjem.18033

**Published:** 2024-06-27

**Authors:** Callan Elswick Fockele, Tessa Frohe, Owen McBride, David L. Perlmutter, Brenda Goh, Grover Williams, Courteney Wettemann, Nathan Holland, Brad Finegood, Thea Oliphant-Wells, Emily C. Williams, Jenna van Draanen

**Affiliations:** * University of Washington, Department of Emergency Medicine, Seattle, Washington; † University of Washington, Department of Psychiatry and Behavioral Sciences, Seattle, Washington; ‡ University of Washington, Department of Health Systems and Population Health, Seattle, Washington; § Research with Expert Advisors on Drug Use, Seattle, Washington; ∥ Public Health – Seattle & King County, Seattle, Washington; ¶ Health Services Research & Development Center of Innovation for Veteran-Centered and Value-Driven Care, Veterans Affairs Puget Sound Health Care System, Seattle, Washington; # University of Washington, Department of Child, Family, and Population Health Nursing, Seattle, Washington

## Abstract

**Introduction:**

Recent policy changes in Washington State presented a unique opportunity to pair evidence-based interventions with first responder services to combat increasing opioid overdoses. However, little is known about how these interventions should be implemented. In partnership with the Research with Expert Advisors on Drug Use team, a group of academically trained and community-trained researchers with lived and living experience of substance use, we examined facilitators and barriers to adopting leave-behind naloxone, field-based buprenorphine initiation, and HIV and hepatitis C virus (HCV) testing for first responder programs.

**Methods:**

Our team completed semi-structured, qualitative interviews with 32 first responders, mobile integrated health staff, and emergency medical services (EMS) leaders in King County, Washington, from February–May 2022. Semi-structured interviews were recorded, transcribed, and coded using an integrated deductive and inductive thematic analysis approach grounded in community-engaged research principles. We collected data until saturation was achieved. Data collection and analysis were informed by the Consolidated Framework for Implementation Research. Two investigators coded independently until 100% consensus was reached.

**Results:**

Our thematic analysis revealed several perceived facilitators (ie, tension for change, relative advantage, and compatibility) and barriers (ie, limited adaptability, lack of evidence strength and quality, and prohibitive cost) to the adoption of these evidence-based clinical interventions for first responder systems. There was widespread support for the distribution of leave-behind naloxone, although funding was identified as a barrier. Many believed field-based initiation of buprenorphine treatment could provide a more effective response to overdose management, but there were significant concerns that this intervention could run counter to the rapid care model. Lastly, participants worried that HIV and HCV testing was inappropriate for first responders to conduct but recommended that this service be provided by mobile integrated health staff.

**Conclusion:**

These results have informed local EMS strategic planning, which will inform roll out of process improvements in King County, Washington. Future work should evaluate the impact of these interventions on the health of overdose survivors.

Population Health Research Capsule What do we already know about this issue?

*First responders have not historically offered harm reduction services that are known to reduce overdose death and increase access to care for people who use drugs.*

What was the research question?

*What are the facilitators and barriers for first responders to provide harm reduction services in the field?*

What was the major finding of the study?

*Perceived facilitators were tension for change, relative advantage, and compatibility, while barriers were limited adaptability, lack of evidence,and prohibitive cost.*

How does this improve population health?

*Participants experienced a tension for change and were activated to implement leave-behind naloxone, field-based buprenorphine, and HIV and hepatitis C virus testing.*

## INTRODUCTION

The public health crisis of opioid use disorder (OUD) and opioid overdose continues unabated, with rates continuing to rise.^1^^–^^3^ Survivors of non-fatal overdose have a significantly greater risk of repeat overdose and overdose-related mortality within the following year, emphasizing the importance of first responder interventions.^4^^–^^7^ These trends are mirrored locally in King County, Washington, where the annual 9-1-1 call volume of probable overdoses and other opioid use-related incidents increased by more than 20% from 2018–2021.^8^ A critical window for intervention exists, as approximately 40% of individuals who died of an overdose in 2018 had at least one emergency medical services (EMS) encounter during the preceding year.^9^

Recent legislative changes in Washington State presented a unique opportunity to pair evidence-based interventions with first responder services to address the rise in opioid overdoses. Specifically, in February 2021, the Washington State Supreme Court struck down the statute that made possession of controlled substances a class C felony. The state government responded by passing a temporary law that expanded the role of first responders (eg, firefighters, paramedics, and police officers) to connect adults found with small amounts of controlled substances to case management instead of the criminal legal system.^10^ In 2023 the legislature rolled back some of these changes with a permanent bill that increased criminal penalties for drug possession and public use and made pre-trial diversion to treatment programs contingent on the prosecutor’s consent.^11^

While first responders have historically provided important referrals to community resources,^12^ such programs have not historically offered harm-reduction resources or treatment initiation. Specifically, there are three medical services that are known to reduce overdose death and increase access to care for people who use drugs: leave-behind naloxone^13^^,^^14^; field-based initiation of buprenorphine treatment^14^^–^^19^; and HIV and hepatitis C virus (HCV) testing.^20^ These interventions have documented efficacy in emergency departments^13^^,^^15^ and community clinics^14^^,^^20^ while demonstrating promising results during brief encounters with street medicine teams and paramedics.^16^^–^^19^ In particular, the distribution of naloxone kits is cost effective^21^^,^^22^ and significantly reduces opioid-related fatalities.^23^^–^^25^ Buprenorphine treatment for OUD may decrease all-cause and opioid-related mortality by up to 50%,^26^^–^^29^ and HIV and HCV testing improves access to care for people who use drugs.^30^ However, there is a paucity of literature on the implementation of these three evidence-based programs in first responder systems.

Grounded in community engaged research (CEnR) principles,^31^ our team partnered with the Research with Expert Advisors on Drug Use (READU), a group of academically trained and community-trained researchers with lived and living experience of substance use, to address this gap. The primary objective was to examine the facilitators and barriers to the adoption of leave-behind naloxone, field-based initiation of buprenorphine treatment, and HIV and HCV testing for first responder programs. The secondary objective was to inform local EMS overdose response policy and programming.

## METHODS

### Study Design and Setting

From March–June 2022, we conducted 32 semi-structured interviews with first responders, mobile medical clinicians, and EMS leaders working in King County, Washington. The study was approved by the University of Washington Institutional Review Board.

### Theoretical Framework

This study was informed by the Consolidated Framework for Implementation Research (CFIR).^32^ By providing a consistently applied set of analytical categories, consisting of “constructs” situated within “domains,” the CFIR^32^ simplifies processes, highlights barriers, and identifies potential areas of improvement (Figure). As described below, this framework provided the scaffolding for the interview guides, deductive coding, and thematic analysis, which highlighted various constructs as perceived facilitators (ie, tension for change, relative advantage, compatibility) and barriers (ie, adaptability, evidence strength and quality, and cost).

**Figure.**
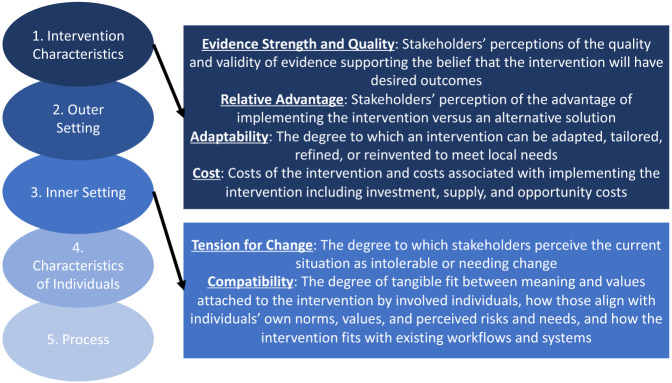
Adapted Consolidated Framework for Implementation Research (CFIR) with numbered domains and selected constructs.

### Reflexivity and Partnership

Our study team was composed of harm reductionists, including both academically trained researchers with advanced degrees in public health, psychology, and medicine, and community-trained researchers with lived and living experience of drug use and EMS system involvement. Together, we embraced CEnR principles,^31^ practiced reflexivity,^33^ and centered the perspectives of people who use drugs in the study’s design, execution, and analyses. Prior to starting data collection, we engaged in bidirectional training during which community-trained READU members educated the academically trained researchers on effective outreach strategies and experiences with past studies, while academically trained researchers shared knowledge about qualitative study design and analysis.

### Participant Recruitment

Participants were recruited through convenience and snowball sampling. We emailed recruitment materials to leaders and administrators at a variety of first responder agencies in King County to disseminate information to potential participants, including paramedics, firefighters, police officers, mobile integrated health staff (ie, co-responding social workers and firefighters engaged in community paramedicine), and mobile medical clinicians (ie, social workers, nurses, physician assistants, and nurse practitioners performing street outreach). Interested individuals contacted the study team through our study phone or email, and they were screened for eligibility. Inclusion criteria included experience working as a first responder, a mobile medical clinician, or in a management/leadership position in a first responder organization in King County; being over 18 years of age; and speaking English.

### Data Collection

Demographic information collected from participants included age, gender, race and/or ethnicity, employment, and highest level of educational attainment. Separate but related interview guides informed by the CFIR^32^ framework were developed for first responders, mobile medical clinicians, and EMS leaders. Topics covered in the interviews included participants’ perceived role within the opioid epidemic; perceptions of services provided to people who use drugs; and the perceived feasibility, acceptance, and appropriateness of leave-behind naloxone, field-initiated buprenorphine, and HIV and HCV testing. The interview guides were iteratively refined, and the final guides are included as an appendix. An academically trained researcher with prior experience in qualitative methods was paired with a community trained READU member to conduct each interview.

### Thematic Analysis

We used an integrated deductive and inductive thematic approach^34^^,^^35^ to analysis. Once the initial interviews were completed, we familiarized ourselves with the data, reviewed the transcripts for accuracy, and noted initial impressions together. We grouped emergent observations into inductive codes and situated them in our preliminary codebook with the pre-existing deductive CFIR codes.^32^ We applied the codebook to a single interview transcript, engaged in line-by-line coding as a group, and reconciled any disagreements in code applications to finalize the codebook. Individual team members then primarily applied the revised codebook to each transcript, and another conducted secondary coding, addressing any differences.

Subsequent semi-structured interviews were conducted until thematic saturation was reached. Interviews were recorded, transcribed, deidentified, uploaded to the qualitative data management software Dedoose (SocioCultural Research Consultants, LLC, Manhattan Beach, CA), and coded deductively using existing CFIR codes^32^ and inductively using codes created from reviewing a sample of transcripts.^36^ We summarized coded data to identify barriers and facilitators to adopting leave behind naloxone, field-based buprenorphine initiation, and HIV and HCV testing for first responder programs, and we extracted prototypical examples of each.

## RESULTS

### Participant Demographics

We interviewed 32 first responders, mobile medical clinicians, and EMS leaders who worked in seven different cities located in King County, Washington (Table 1). Participants included Basic Life Support professionals (ie, firefighter/emergency medical technicians), Advanced Life Support professionals (ie, paramedics), police officers, nurses, and advanced registered nurse practitioners, social workers, and EMS leaders. Of the first responders interviewed, 19 (59%) had been in their current role for more than 10 years. Participants were 31.3% female and 12.5% racially/ethnically diverse, and most were above the age of 36 with at least some college education.

**Table 1.** Interviewees’ demographic information.

Age	n (%)
20–25	2 (6.3%)
26–35	5 (15.6%)
36–45	11 (34.4%)
46–55	6 (18.8%)
56–65	8 (25%)
Gender	n (%)
Male	20 (62.5%)
Female	10 (31.3%)
Trans, non-binary, or gender non-conforming	2 (6.3%)
Race and/or ethnicity	n (%)
White	28 (87.5%)
Asian or Pacific Islander	2 (6.3%)
Hispanic	1 (3.1%)
Mixed race	1 (3.1%)
Employment	n (%)
Basic Life Support professionals (ie, firefighter/emergency medical technicians)	8 (25%)
Advanced Life Support professionals (ie, paramedics)	6 (18.8%)
Police officers	5 (15.6%)
Nurses and advanced registered nurse practitioners	3 (9.4%)
Social workers	5 (15.6%)
Emergency medical services leaders	5 (15.6%)
Number of years in current role	n (%)
<1	2 (6.3%)
1–4	8 (25%)
5–9	3 (9.4%)
10–19	8 (25%)
>20	11 (34.4%)
Highest level of educational attainment	n (%)
Associate’s degree	8 (25%)
Bachelor’s degree	8 (25%)
Master’s degree	10 (31.3%)
Doctoral degree	2 (6.3%)
Unspecified	4 (12.5%)

### Qualitative Results

Through the lens of the CFIR framework,^32^ our thematic analysis revealed several perceived facilitators (ie, tension for change, relative advantage, and compatibility) and barriers (ie, limited adaptability, lack of evidence strength and quality, and prohibitive cost) to the adoption of three evidence-based clinical interventions for first responder systems: 1) leave behind naloxone; 2) field-based initiation of buprenorphine treatment; and 3) HIV and HCV testing.

### Leave-behind Naloxone

There was widespread support for the distribution of leave-behind naloxone with many acknowledging a tension for change and finding the intervention relatively advantageous and compatible within existing systems (Table 2). Many interviewees recognized that naloxone is a safe, easy-to-use, indispensable medication that should be accessible to patients, their loved ones, and other community responders. Implementation of leave- behind naloxone was also largely thought to be feasible with several interviewees explaining that distribution could be effortlessly integrated into current workflows.

**Table 2.** Interviewees’ perceived facilitators and barriers to implementing a leave-behind naloxone program.

Facilitators	
Tension for change	“And I think, yes, certainly the fire department should play a role in having access to that and being able to hand it out and providing education on how to use it and when to use it.”—Paramedic (ID #25)
Relative advantage	“I think that naloxones are [a] lifesaving intervention, and it’s relatively easy for people to administer to their friends or bystanders can administer to people they don’t know. So, I do think naloxone is very important and it should be out there and there should be access to it. And us leaving it behind with people, I think is a good idea.”—Paramedic (ID #7)
Compatibility	“I think that’s probably the easiest one … We could absolutely get the Narcan … First responders definitely can provide [those] as an intervention.”—Mobile integrated health social worker (ID #20)
**Barriers**	
Limited adaptability	“I feel like it’d be a psychological thing for officers, especially officers who’ve been around for 10 plus years, where we used to arrest drug dealers and put them in jail. And now we’re ignoring the crimes they’re committing and we’re giving them naloxone so that they can further just continue to use drugs. So, I can see someone who is maybe not looking at the full picture or just has their personal beliefs.”—Police officer (ID #1)
Lack of evidence strength and quality	“I worry that we’re just put[ting] more people in withdrawal and sort of miss[ing] the opportunities to do something about it.”—Interviewee in leadership or management role (ID #28)
Prohibitive cost	“But I also have some skepticism that sort of just throwing out naloxone kits is gonna make a big difference. I’m not opposed to it, but it does require more effort and time and energy, and there’s a cost to it. And quite frankly, we have [a] limited budget, and so, who’s going to pay for those things? I don’t know. So I’m measured in my support for that program, but if there’s evidence that it saves lives, then we will work towards that.”—Interviewee in leadership or management role (ID #27)

A smaller group of individuals expressed concern about potential barriers, particularly limited adaptability, lack of evidence strength and quality, and prohibitive cost. Some police officers thought that naloxone distribution may encourage unsafe behaviors (eg, using larger amounts or more potent substances) and felt that it was incongruous with their departments’ current approach to controlling drug use through legal penalties and incarceration. Other service professionals worried that increased access to naloxone would lead to community members, rather than first responders, managing more overdose responses and consequently decreasing the likelihood of connecting people to treatment and other resources. Lastly, several interviewees in leadership or management roles were skeptical about the relative benefit of naloxone, explaining that they believed there ought to be more evidence on the efficacy of leave-behind naloxone programs. They also worried about the resources and training required for implementation.

#### Field-based Initiation of Buprenorphine Treatment

Despite having less familiarity with the medication compared to naloxone, most interviewees recognized a tension for change and approved of the implementation of field-based initiation of buprenorphine treatment, considering it evidence-based, appropriate, and relatively advantageous for their settings (Table 3). Many felt unprepared to address withdrawal, particularly when a patient’s overdose may have been fully reversed with bystander naloxone, but buprenorphine was seen as a “destigmatizing” tool that relieves symptoms, demonstrates compassion, and builds trust between patients and first responders. Additionally, participants described how the recent uptick in overdose responses, occasionally with the same individuals, led to burnout and a desire to address the upstream causes of substance use. Several highlighted how field-based initiation of buprenorphine treatment could bridge vulnerable individuals to ongoing treatment, potentially preventing future overdoses, decreasing overall call volumes, and saving lives.

**Table 3.** Interviewees’ perceived facilitators and barriers to field-based initiation of buprenorphine treatment.

Facilitators	
Tension for change	“I think the opioid issue that we have in our kind of city right now, it’s big and it takes a big toll on people. And I think that if there is evidence that shows that Suboxone or buprenorphine can help, and … especially if we’re following in the footsteps of another agency or agencies that have used it and have some data on what works and what doesn’t, then I would be all for it.”—Mobile medical nurse (ID #15) “Suboxone is good stuff. If we’re truly trying to help people transition out of addiction, it’s a great tool to help manage withdrawals. As far as in the field, I think if we could provide them access to it, absolutely, I would be 100% behind that.”—Firefighter (ID #4) “I think EMS is often the first interaction of a pretty traumatic chain of events leading to the ED. And so, I think if that engagement were positive, there’d be less hesitation to call 911, number one, for overdose. And then number two, every chance we can give someone to decrease or stop their opioid use is well worth it. It feels a little more like we’re making a difference than giving the naloxone, the Narcan, 'cause here it’s like, ‘This is going to help you wean your body off this stuff.’"—Mobile medical social worker (ID #11)
Relative advantage	“I would say, absolutely any way that we can expand our reach to our community and get them more support, and for addictions and for recovery, I would think would be optimal. And I think that the fire service is a great way to allow that to happen … I’m in full support. I think that would be advantageous in our community.”—Paramedic (ID #25) “And it seems far more of a viable option to me than the leave at home [naloxone]. So the [leave behind naloxone] was just gonna solve the problem in the minute. But it does not take away the next problem, which is I need more, whereas buprenorphine does address that … But the better option [is] to how to get that medicine to people.”—Interviewee in leadership or management role (ID #28)
**Barriers**	
Limited adaptability	“That would be potentially good… [But] we’re [a] busy unit … how much out of service time would that add to the unit to do that?”—Paramedic (ID #22)
Lack of evidence strength and quality	“We’ve made life easier for all these [people who use drugs] out in Seattle, and it hasn’t made things better. It’s actually made things worse. I mean, we’re looking at like 270 deaths so far just in this first quarter. That is four times more than three or four years ago. So, I don’t know if giving suboxone is actually helpful.”—Police officer (ID #1)

*EMS*, emergency medical services; *ED*, emergency department.

Those opposed were largely concerned with this intervention’s limited **adaptability** to the rapid service delivery model of emergency services, emphasizing that the time needed for the intervention may overburden an already overwhelmed system. However, others suggested that the deployment of specialized teams (eg, mobile integrated health or mobile medical clinic teams) dedicated to treating this patient population may be a way to offset these demands. Finally, some police officers worried about the **evidence strength and quality** of buprenorphine, speculating that it could be diverted for non-prescribed use and could encourage ongoing risky behaviors by curbing withdrawal symptoms.

### HIV and Hepatitis C Virus Testing

Interviewees observed the tension for change in their organizations and generally supported increasing access to HIV and HCV testing (Table 4). Some felt that first responder encounters could serve as relatively advantageous opportunities to engage individuals who may not feel comfortable seeking care in more traditional settings. Providing HIV and HCV testing in a trauma-informed manner was seen to increase education around prevention and improve linkage to care.

**Table 4.** Interviewees’ perceived facilitators and barriers to HIV and hepatits C virus testing.

Facilitators	
Tension for change	“This is one of those things that is in our realm of … responsibility. Our primary goal is to help people with what’s happening right now, but if we can also help them out with like, ‘Well, what is the next step for you?’”—Mobile integrated health social worker (ID #17)
Relative advantage	“Hundred percent like the idea of being able to have an agency that has a contract that this is what they do. You go out, and you provide somebody an HIV test. We have people that are specially trained to deal with all the ramifications of somebody who finds out they have HIV, 'cause that’s gonna be a horrible feeling.”—Firefighter (ID #4)
**Barriers**	
Limited adaptability	“That wouldn’t be something useful for first responders because our priority is not necessarily testing and trying to diagnose whether individuals have [a] specific disease.”—Firefighter (ID #2) “I just think that’d be horrible to do to somebody … Like HIV or hepatitis C, like those are huge things. So, you just don’t want to just drop a bomb on somebody on top of them being … During a drug overdose, for example.”—Paramedic (ID #25)

Many, however, were concerned about the adaptability, appropriateness, and feasibility of HIV and HCV testing during an EMS response. Some worried that it would be inconsistent with the rapid service delivery model of emergency services since point-of-care testing takes at least 20 minutes to complete.^37^^,^^38^ Others voiced that testing may feel compulsory and coercive if completed immediately after an unnerving overdose event. Like field-based buprenorphine starts, some interviewees alternatively proposed having first responders hand off these patients to a specialized team that would have more time to conduct the tests, provide the appropriate counseling, and arrange follow-up as needed for confirmatory diagnosis and treatment.

## DISCUSSION

Working on the frontlines of the opioid epidemic, first responders, mobile medical clinicians, and EMS leaders are confronted with skyrocketing overdose responses. Many want to improve the care of patients who use drugs, beyond acute overdose reversal, but feel uncertain about how to proceed. People who use drugs have also expressed a need for improved care with many refusing EMS transport following overdose due to law enforcement’s presence at overdose scenes,^39^ unmanaged withdrawal symptoms, and anticipated stigmatizing treatment by EMS and emergency clinicians.^40^ Our thematic analysis informed by the CFIR framework^32^ identified several perceived facilitators (ie, tension for change, relative advantage, and compatibility) and barriers (ie, limited adaptability, lack of evidence strength and quality, and prohibitive cost) to the adoption of three evidence-based clinical interventions for first responder systems: 1) leave-behind naloxone; 2) field-based initiation of buprenorphine treatment; and 3) HIV and HCV testing. However, there are few examples of implementing these evidence-based interventions in first responder systems with one narrative review finding only 27 programs out of nearly 22,000 EMS agencies nationally described in the literature, with many providing naloxone distribution and community referrals while few facilitated linkage to medications for OUD.^41^

Many recognized the tension for change in their community and the relative advantage of distributing naloxone kits and treating OUD with buprenorphine in the field. Leave-behind naloxone is a cost-effective,^21^^,^^22^ widely accepted^42^^–^^44^ tool that reduces opioid overdose-related mortality^45^^,^^46^ and does not increase risky drug use behavior.^47^ Existing EMS programs distributing naloxone kits demonstrated feasibility^48^ and increased connection to other resources.^49^ Most interviewees believed leave-behind naloxone was compatible with and could be easily integrated into their workflows, yet several highlighted the importance of securing sustainable funding to address costs and receiving additional training to address the perceived lack of evidence strength and quality before implementation. Participants were similarly enthusiastic about the prospect of treating opioid withdrawal and OUD with buprenorphine. In addition to an initial case series describing treating withdrawal from naloxone administration with buprenorphine,^18^ a pilot study examining prehospital buprenorphine treatment for OUD showed 50% retention in treatment at seven days and 36% in 30 days.^19^

Notably, participants working in law enforcement were more skeptical of harm reduction than those employed in healthcare and social services. Some expressed frustration with recent legislation that curtailed criminal penalties for drug possession and public use. Other law enforcement officers expressed sentiments similar to those of healthcare and social services workers but questioned what their role in addressing the opioid epidemic could be under the new laws. Importantly, police officers still regularly respond to medical emergencies involving drug use, including overdoses, highlighting the urgent need for targeted education on how to use these evidence-based interventions effectively in the field.

Lastly, the most discussed barrier to all three interventions, particularly field-based initiation of buprenorphine and HIV and HCV testing, was a feeling from frontline professionals that implementation had limited adaptability to the rapid service delivery model of emergency services. However, others recommended either deploying a specialized team to the scene or transporting the patient to a diversion facility that could provide wraparound services. Local mobile medical clinic teams have successfully integrated harm reduction services into their care of those experiencing homelessness,^50^ and the creation of mobile integrated health response units have expanded case management and referrals through multidisciplinary collaborations in fire departments.^51^ With longer dispatch time and the ability to do longitudinal follow-up, these teams may be well suited to provide post-overdose care.

The Philadelphia Fire Department has an alternative response unit (“AR-2”) equipped with Advanced Life Support capabilities, which is located in an area heavily impacted by opioid overdoses. It responds to those resuscitated with naloxone but who refuse transportation to the hospital, and early data demonstrates that 84% of patients accepted services, including treatment facility placement, resources, and/or naloxone kits.^52^ Diversion facilities offering low-barrier access to treatment and other services could also operate as an alternative to a prolonged EMS response or emergency department visits; in fact, a former hospital facility in Columbus, Ohio, now equipped with 60 beds dedicated to addiction stabilization serves as the primary post-overdose receiving center for individuals seeking treatment and deemed medically stable by EMS.^53^

## LIMITATIONS

Our objective in this study was to examine the facilitators and barriers to the adoption of leave-behind naloxone, field-based initiation of buprenorphine treatment, and HIV and HCV testing for first responder programs. However, the results may only be applicable to the geographic location of the interviewees, which included first responders, mobile medical clinicians, and EMS leaders working in King County, Washington. Racial and ethnic minorities were notably poorly represented in our study. Because there is no publicly available data on the demographic information of EMS professionals locally, we were unable to assess whether our sample was representative. Our convenience and snowball sampling may have also introduced bias. Most participants described being in their current role for more than 10 years, which is likely much higher than the general first responder population. Finally, we did not track the decline-to-be interviewed rate.

## CONCLUSION

Without the tools to address the uptick in opioid overdoses, first responders, mobile medical clinicians, and EMS leaders in King County experienced a tension for change and are now activated to implement leave- behind naloxone, field-based initiation of buprenorphine treatment, and HIV and HCV testing through new EMS protocols, post-overdose response teams, and diversion facilities. In this study we took a team-based approach and centered the perspectives of people with lived and living experience of drug use to ensure that this research led to action. Members of READU highlighted our work’s relevance to the community and framed these findings to inform policy, particularly with the recent changes in Washington State legislation. Future works should evaluate the impact of these interventions on the health of overdose survivors.
